# Persistent Cortical Blindness Following Posterior Reversible Encephalopathy Syndrome (PRES) as a Complication of COVID-19 Pneumonia

**DOI:** 10.7759/cureus.12794

**Published:** 2021-01-19

**Authors:** Mohamed Elhassan, Ola Saidahmed, Ayobami Adebayo, Neil Archibald

**Affiliations:** 1 Cardiology, Royal Derby Hospital, Derby, GBR; 2 Obstetrics and Gynaecology, University Hospitals of Derby and Burton NHS Foundation Trust (UHDB), Derby, GBR; 3 Internal Medicine, South Tees Hospitals NHS Foundation Trust, Middlesbrough, GBR; 4 Neurology, South Tees Hospitals NHS Foundation Trust, Middlesbrough, GBR

**Keywords:** covid-19, sars-cov-2, posterior reversible encephalopathy syndrome (pres), anton's syndrome, cortical blindness

## Abstract

The novel severe acute respiratory syndrome coronavirus 2 (SARS-CoV-2) pandemic emerged in China in December 2019. Since then, there have been growing reports of coronavirus disease 2019 (COVID-19) cases with neurological involvement. We present a case of a 54-year-old woman who presented with confirmed SARS-CoV-2 pneumonia, complicated by a prolonged intensive care stay and posterior reversible encephalopathy syndrome (PRES). This resulted in persistent cortical blindness (Anton’s syndrome). PRES has only rarely been reported in relation to SARS-CoV-2 infection and no patients have developed persistent cortical blindness. We summarise the clinical presentation of the patient and review the current literature.

## Introduction

Since the emergence of the severe acute respiratory syndrome coronavirus 2 (SARS-CoV-2) pandemic in China, in December 2019, there has been growing reported literature suggesting a variety of neurological manifestations and complications [1]. These include neurological symptoms at presentation, such as headache, dizziness, encephalopathy, delirium, olfactory and gustatory disorders and subsequent complications, including cerebrovascular accidents, meningitis, Guillain-Barré syndrome, acute transverse myelitis, and encephalitis [1,2]. Emerging evidence suggests that the underlying mechanisms for these manifestations include direct viral invasion or maladaptive inflammatory responses [2].

In this report, we present a case of posterior reversible encephalopathy syndrome (PRES), complicated by Anton’s cortical blindness, in the presence of severe SARS-CoV-2 pneumonia. This is one of a few reported cases of PRES as a complication of severe SARS-CoV-2 pneumonia and the first to result in persisting cortical blindness.

This article was previously presented as a meeting abstract at the 2020 UK Stroke Forum Annual Scientific Meeting on December 7, 2020.

## Case presentation

A previously fit and well 54-year-old woman presented with a 10-day history of fever, dry cough, and myalgia. She also reported worsening breathlessness two days before presentation. Her admission observations were as follows: temperature 38.2 °C, pulse 114 bpm, and blood pressure 125/78 mmHg. Her initial oxygen saturations were 82% on room air. Following further clinical deterioration, and continuing increasing oxygen requirements, she was started on non-invasive ventilation (with initial positive end-expiratory pressure (PEEP) of 10 cmH2O and fraction of inspired oxygen (FiO2) of 60% - as per local protocol).

Investigations demonstrated lymphopenia (0.9 x10^9/L) with an otherwise normal complete blood count, normal renal function, normal electrolytes, normal coagulation profile, and mildly raised C-reactive protein (CRP) at 16. Her ECG showed sinus tachycardia. Chest X-ray confirmed bi-basal consolidation. Nasopharyngeal swab for SARS-COV-2 polymerase chain reaction (PCR) was positive. She was started on treatment with amoxicillin/clavulanic acid and clarithromycin empirically.

Her oxygen requirements continued to increase (with persisting high PEEP requirements) and, on day 10 after admission, she underwent endotracheal intubation and mechanical ventilation. Acute respiratory distress syndrome (ARDS) was diagnosed. A diagnosis of systemic inflammatory response syndrome (SIRS) was made five days later (the patient had fever and tachycardia), with no clear source or clinical focus of infection. The CRP was around 300 and leucocyte count was 16 x10^9/L. Multiple blood and urine cultures were negative. As sepsis was difficult to rule out, empirical, broad-spectrum antibiotics were started, to good effect. She did not receive any steroids, immunomodulatory, biologic nor immunosuppressive therapy throughout admission.

**Figure 1:**
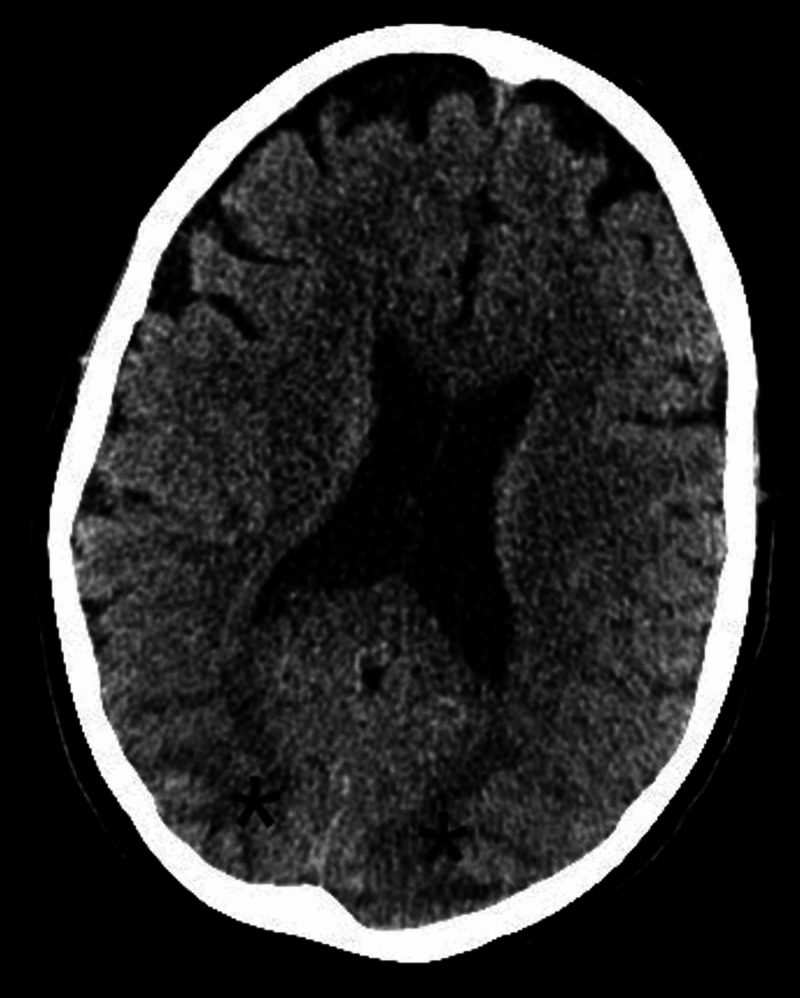
CT scan of the head showing evidence of relative hypodensities (marked with asterisks) suggestive of posterior reversible encephalopathy syndrome (PRES)

On day 21 of admission, she had a self-terminating generalised tonic-clonic seizure. The blood pressure was briefly raised to 190/90 mmHg after this episode but was otherwise maintained in the normal range (165-121 mmHg systolic and 69-62 mmHg diastolic). A second generalised seizure occurred the following day. A CT scan of the head was done immediately after this episode. This showed posterior changes suggestive of PRES (Figure 1).

Over the next 10 days, her condition stabilised and she underwent extubation. Neurological examination revealed complete cortical blindness, with no perception of light. Pupil responses to light were normal and fundoscopy unremarkable. She had poor insight into the extent of her visual impairment, often claiming to be able to see (Anton’s syndrome). She also described visual hallucinations. The patient had a mild receptive and more marked expressive dysphasia as well as evidence of limb apraxia, most marked on the right side.

Over the next eight weeks, she made a gradual physical and neurological recovery. She was able to make out shapes and colours but remained profoundly sight-impaired.

**Figure 2:**
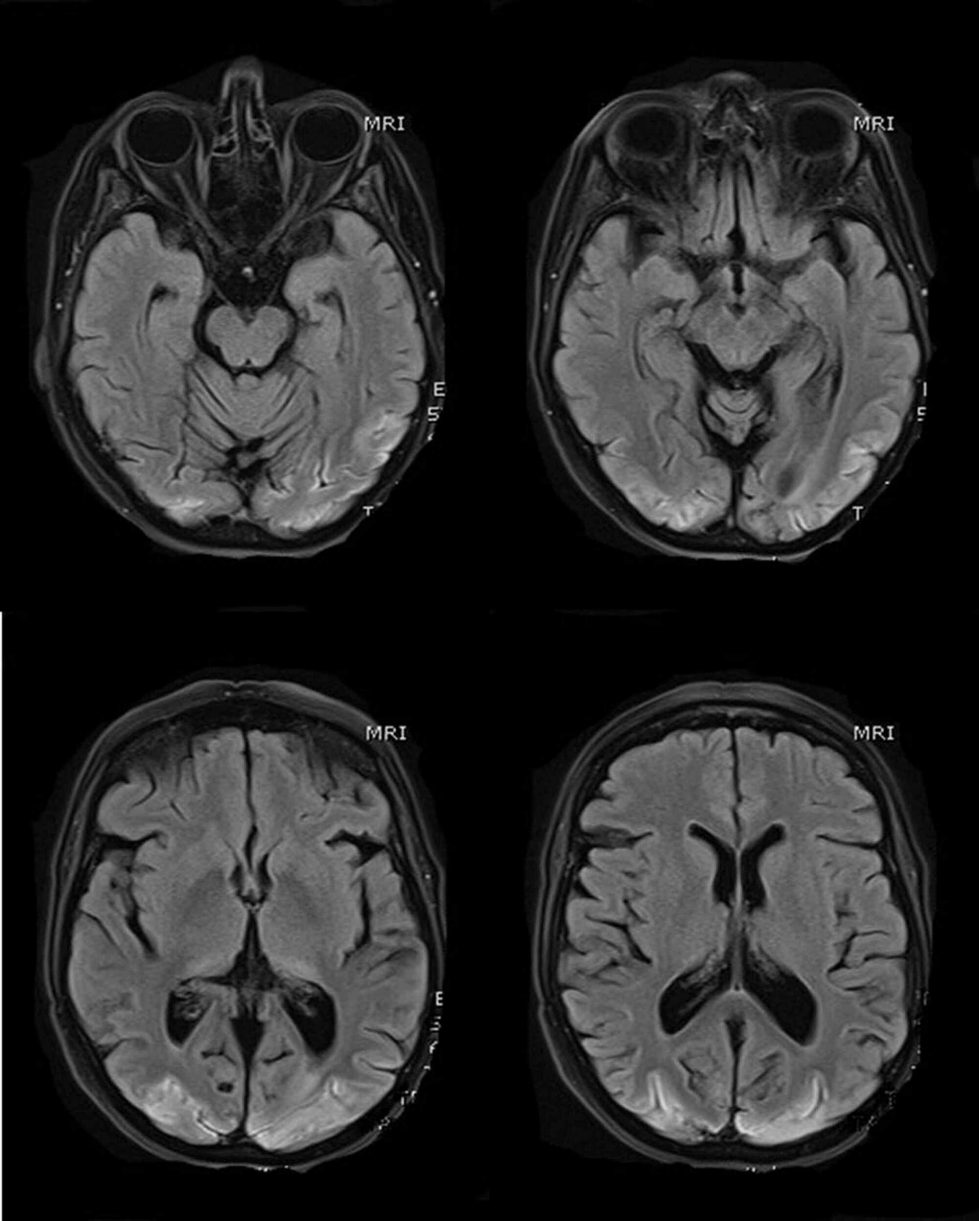
MR scans of the brain (selected T1 sequences) show bilateral symmetrical hyper-intensity involving the bilateral occipital lobe cortex; the changes are consistent with cortical pseudo-laminar necrosis as a complication of posterior reversible encephalopathy syndrome (PRES)

MR scan of the brain performed nine weeks after her admission demonstrated changes consistent with hypoxic-ischemic encephalopathy secondary to PRES (Figure 2).

## Discussion

PRES is a neurovascular syndrome characterised by acute onset headache, seizures, focal neurological deficits, disturbed consciousness, and visual symptoms. Brain imaging characteristically shows evidence of posterior cerebral white matter oedema. Risk factors for PRES include hypertension and hypertensive encephalopathy, renal disease, certain immunosuppressive and immunomodulatory therapy, some chemotherapeutic medications, sepsis, and others [3].

The cause of PRES remains unclear with controversy regarding underlying causative pathophysiological mechanisms [4]. The two main theories include the hypertensive brain hyper-perfusion theory (with autoregulation failure) and the endothelial dysfunction theory [4].

The diagnosis of PRES, in our case, was suggested by the clinical and radiological findings. Although MR scans were neither feasible nor safe at the time of diagnosis period, a CT scan of the brain showed evidence of occipital subcortical involvement suggestive of PRES. Follow up imaging with a different modality around six weeks later (MRI) showed resolution of the subcortical changes and establishment of cortical pseudo-laminar necrosis as a complication of PRES. Indeed, the time interval for radiologic resolution of PRES features ranges between two to four weeks but can be as low as eight days [3,5,6].

Kishfy et al. reported two cases of PRES occurring in relation to SARS-CoV-2. As in our patient, PRES occurred with only relatively modest blood pressure fluctuations. Unlike our case, there was no visual disturbance. The authors have suggested that tighter blood pressure control might be required in coronavirus disease 2019 (COVID-19) patients to reduce the risk of PRES [7].

Our patient’s admission was complicated by possible sepsis, a known risk factor for PRES, and this raises the possibility that, aside from blood pressure changes, endothelial dysfunction was a contributory factor in this case [3]. Indeed, there is some evidence that SARS-CoV-2 binds to angiotensin-converting enzyme 2 (ACE2) receptors, which may suggest a direct pathological role in endothelial dysfunction [8]. It is possible that the co-existence of several risk factors might have a synergistic effect in the causation of PRES. Also, there is a possibility that the severe course of COVID-19 illness in our case, especially with the negative culture results, was the cause of sepsis per se. 

In our case, the highest blood pressure recorded was 190/90 mmHg. This surge happened only after the first seizure activity and is likely secondary to the seizure itself [9]. Otherwise, the blood pressure was within normal limits throughout. Furthermore, the fact that the patient was never known to have hypertension suggests that this aetiology is not the primary cause for PRES in our case. 

Kaya et al. also reported a case of COVID-19 that resulted in a PRES-like picture. This case resulted in transient cortical blindness. The authors reported that they were not able to identify an aetiology for the development of PRES although the blood pressure was reported to be transiently elevated at the time of neurological symptom onset [10].

## Conclusions

This case, along with the aforementioned cases, suggests that PRES could be triggered by severe COVID-19 infection. Hence, severe illness with a risk factor for PRES and acute onset of blindness in a COVID-19 patient may warrant neurologic imaging and PRES should be included in the differential. Also, there might be a significant association between SARS-CoV-2 infection and endothelial dysfunction in the pathogenesis of PRES. Lastly, the occurrence of PRES with relatively narrow blood pressure fluctuations might suggest the need for tighter blood pressure control overall.
